# Retrospective Comparison of Influenza and COVID-19-Associated Acute Respiratory Distress Syndrome (ARDS): An Experience From a Tertiary Care Hospital

**DOI:** 10.7759/cureus.66636

**Published:** 2024-08-11

**Authors:** Rizwana Rana, Aftab Akhtar

**Affiliations:** 1 Critical Care Medicine, Shifa International Hospital, Islamabad, PAK; 2 Pulmonary Medicine, College of Physicians and Surgeons of Pakistan, Islamabad, PAK; 3 Internal Medicine, Shifa International Hospital, Islamabad, PAK

**Keywords:** ace-2 receptor, covid-19 pcr, influenza virus type a and b, airborne infection control, cardio vascular disease, comorbidities, icu admission, critically ill patients, influenza, covid-19

## Abstract

Background

The COVID-19 pandemic has had a profound impact on global healthcare systems, often compared to seasonal influenza due to similarities in clinical presentation. This study aims to compare the clinical characteristics, comorbidities, and outcomes of critically ill patients with COVID-19 and those with influenza admitted to a tertiary care hospital in Islamabad, Pakistan.

Methods

This retrospective cohort study included 120 patients, 60 with confirmed COVID-19 and 60 with confirmed influenza, all of whom required ICU admission and mechanical ventilation between January 1, 2021, and January 1, 2024. Data were collected from electronic medical records, including demographic information, comorbidities, and clinical outcomes. Descriptive statistics were used to compare the two groups.

Results

The median age of COVID-19 patients was 55 years (range 30-78), while that of influenza patients was 58 years (range 31-80). Both groups had a slight male predominance (COVID-19: 66.7%, Influenza: 63.3%). Comorbidities were common in both groups, with 75.0% of COVID-19 patients and 83.3% of influenza patients having at least one comorbidity. The most common comorbidities included hypertension (COVID-19: 30.0%, Influenza: 33.3%) and diabetes (COVID-19: 20.0%, Influenza: 25.0%).

Clinical outcomes revealed a higher mortality rate among influenza patients (43.3%) compared to COVID-19 patients (28.3%). ICU admission rates were identical for both groups at 66.7%, and mechanical ventilation was required for 66.7% of ICU-admitted patients in both groups. The presence of cardiovascular comorbidities significantly impacted patient outcomes, with higher mortality observed in influenza patients with such comorbidities (44.7%) compared to COVID-19 patients (28.9%).

Conclusion

This study highlights the significant burden of both COVID-19 and influenza on critically ill patients, particularly those with cardiovascular comorbidities. While influenza patients in this cohort exhibited higher mortality rates, both groups demonstrated substantial ICU admission rates and a need for mechanical ventilation.

## Introduction

The comparison of clinical features and outcomes in critically ill patients hospitalized with COVID-19 versus influenza has been a topic of interest, especially in the context of patient management and therapeutic interventions. The available literature provides valuable insights into various aspects of critical illness, including the use of systemic corticosteroids, invasive mechanical ventilation, organ support, and specific treatments, such as angiotensin-converting enzyme (ACE) inhibitors, angiotensin receptor blockers (ARBs), and P2Y12 inhibitors. Additionally, the studies highlight the associations between clinical parameters and outcomes, including respiratory outcomes, mortality, and the impact of co-infections.

Sterne et al. conducted a prospective meta-analysis, reporting that the administration of systemic corticosteroids was associated with lower 28-day all-cause mortality in critically ill patients with COVID-19 [1]. Cummings et al. conducted a prospective cohort study and found that critical illness among patients hospitalized with COVID-19 was associated with a high frequency of invasive mechanical ventilation, extra-pulmonary organ dysfunction, and substantial in-hospital mortality [2]. Berger et al. explored the use of P2Y12 inhibitors in non-critically ill hospitalized patients with COVID-19 and found that the addition of P2Y12 inhibitors to therapeutic doses of heparin did not result in increased odds of improvement in organ support-free days within 21 days during hospitalization [3]. Cobb et al. compared the clinical features and outcomes of critically ill patients hospitalized with COVID-19 versus influenza [4]. The study found that patients with COVID-19 had worse respiratory outcomes, longer duration of mechanical ventilation, and greater risk for in-hospital mortality compared to those with influenza. This comparison provides valuable insights into the distinct clinical features and outcomes of COVID-19 and influenza in critically ill patients. Kurihara et al. investigated the clinical characteristics and outcomes of lung transplant in patients with COVID-19-associated acute respiratory distress syndrome (ARDS). The study reported 100% survival in patients who underwent lung transplant, highlighting the potential benefits of this intervention in managing critically ill patients with COVID-19-associated ARDS [5]. Rodríguez et al. reported that the initiation of ACE inhibitors or ARBs did not improve, and likely worsened, clinical outcomes in critically ill adults with COVID-19 [6]. Sulaiman et al. found that delirium was common in critically ill patients with COVID-19 and appeared to be associated with greater disease severity. However, the presence of delirium was not associated with increased mortality, highlighting the need for further research on the impact of delirium in COVID-19 patients [7]. Lenka et al. reported that bacterial and fungal co-infections and superinfections were frequent in COVID-19 patients admitted to the ICU and were associated with worse outcomes [8]. Williamson et al. investigated the association between lung microbiota and non-resolving ARDS in COVID-19 patients. The study found that bacterial and fungal lung microbiota were related to non-resolving ARDS in COVID-19 and represented an important contributor to heterogeneity in COVID-19-related ARDS [9]. Abulmeaty et al. reported that COVID-positive and influenza-negative patients had lower hospital charges, shorter hospital stays, and overall lower mortality, supporting the use of the influenza vaccine in COVID-positive patients [10]. Literature provides valuable insights into the clinical features and outcomes of critically ill patients hospitalized with COVID-19, including the impact of specific treatments, disease severity, and co-infections [1-10]. However, there are knowledge gaps regarding the data from the developing world.

The objective of this study was to compare the clinical characteristics, comorbidities, and outcomes of critically ill patients with COVID-19 and those with influenza who required ICU admission and mechanical ventilation at a tertiary care center in Islamabad, Pakistan.

## Materials and methods

This study is a retrospective cohort analysis conducted at Shifa International Hospital, a tertiary care center in Islamabad, Pakistan. The aim was to compare the clinical characteristics, comorbidities, and outcomes of critically ill patients diagnosed with either COVID-19 or influenza who required ICU admission and mechanical ventilation.

**Figure 1:**
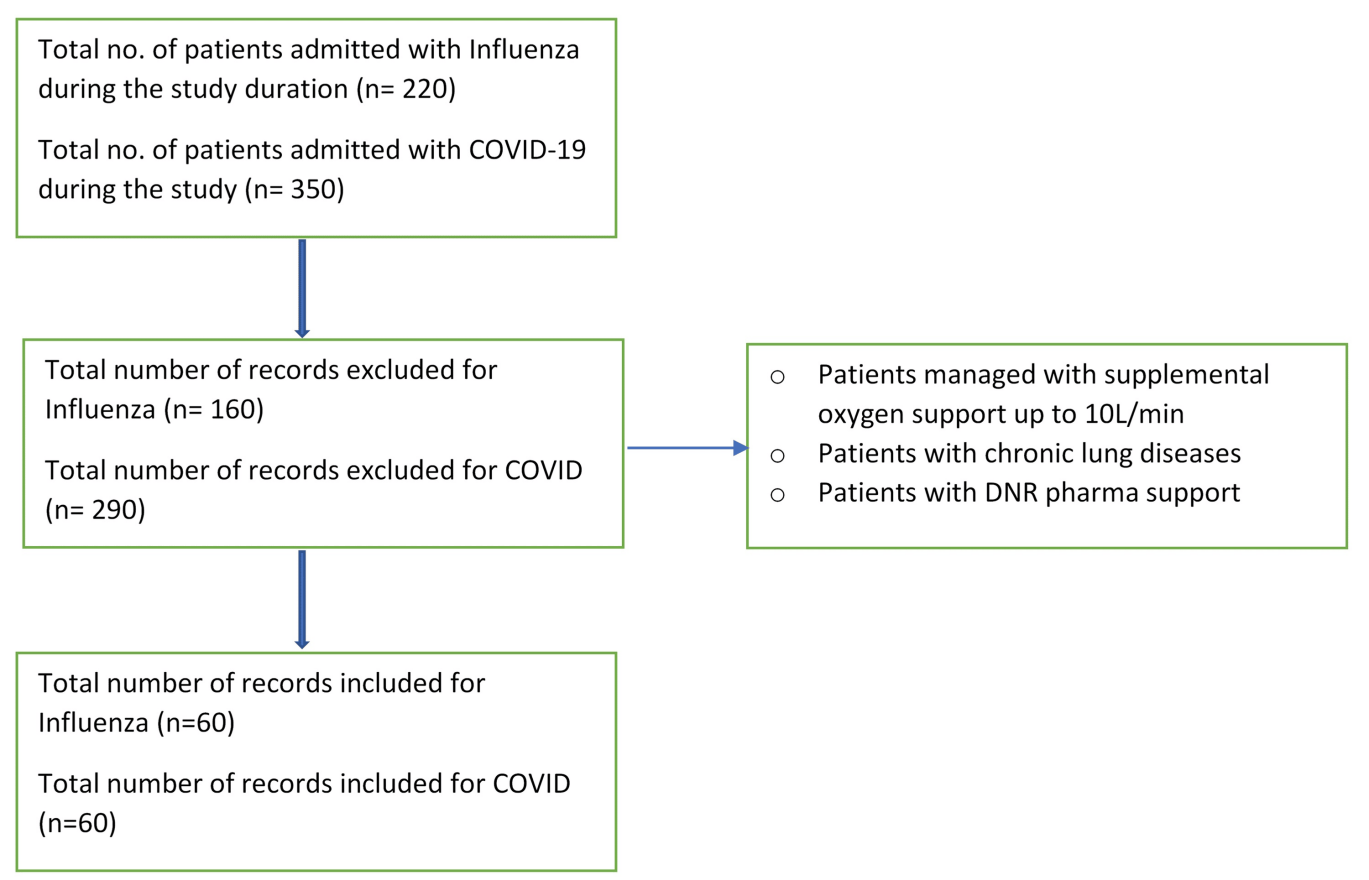
Flowchart for the records included in the study

A total of 220 patients with influenza and 350 patients with COVID-19 required admission to our hospital during the study timeframe. The study excluded 160 patients with influenza and 290 patients with COVID-19-associated ARDS for the reasons outlined in the flowchart below (Figure 1). Each group included 60 patients. All patients were admitted to the ICU between January 1, 2021, and January 1, 2024. COVID-19 diagnosis was confirmed by a positive PCR test for SARS-CoV-2, and influenza diagnosis was confirmed by a positive PCR test for influenza A or B. Data were collected retrospectively from electronic medical records. The extracted information, including age, gender, and comorbidities (presence of hypertension, diabetes, chronic kidney disease, chronic obstructive pulmonary disease (COPD), and asthma), was compared, and clinical outcomes, including mortality, need of ICU admission, need of invasive mechanical ventilation requirement, and rates of prolonged mechanical ventilation (> 7 days), were analyzed.

Data analysis was performed using Python and the Pandas library. Descriptive statistics were calculated for all variables. Continuous variables were reported as median (range) and mean ± standard deviation (SD). Categorical variables were expressed as frequencies and percentages.

The study was conducted in accordance with the ethical standards of the institutional research committee and with the 1964 Helsinki Declaration and its later amendments. Given the retrospective nature of the study, patient consent was waived by the institutional review board.

The statistical analysis and data visualization were conducted using IBM SPSS Statistics for Windows, Version 22 (Released 2013; IBM Corp., Armonk, New York, United States) for creating visual representations of the data. The data was processed and analyzed in a secure, offline environment to ensure patient confidentiality and data integrity.

## Results

A total of 120 patients were analyzed, comprising 60 COVID-19 patients and 60 influenza patients. The median age of the patients in this study was 55 years, with a range from 30 to 78 years for COVID-19 patients, and 58 years, with a range from 31 to 80 years for influenza patients. The mean age for COVID-19 patients was 54.87 ± 13.75 years, while the mean age for influenza patients was 55.65 ± 13.63 years. In terms of gender distribution, the COVID-19 cohort consisted of 40 males (66.7%) and 20 females (33.3%), whereas the influenza cohort included 38 males (63.3%) and 22 females (36.7%). Comorbidities were prevalent in both groups: among COVID-19 patients, 18 (30.0%) had hypertension, 12 (20.0%) had diabetes, eight (13.3%) had chronic kidney disease, five (8.3%) had chronic obstructive pulmonary disease (COPD), and 10 (16.7%) had asthma. Among influenza patients, 20 (33.3%) had hypertension, 15 (25.0%) had diabetes, 10 (16.7%) had chronic kidney disease, seven (11.7%) had COPD, and 12 (20.0%) had asthma (Table 1). Clinical outcomes revealed that ICU admission was required for 40 COVID-19 patients (66.7%) and 40 influenza patients (66.7%). Mechanical ventilation was necessary for 66.7% of ICU-admitted patients in both groups. The mortality rate was 28.3% (17 patients) for the COVID-19 group and 43.3% (26 patients) for the influenza group.

**Table 1:** Demographic characteristics and comorbid profiles of patients included in the study

Characteristic	COVID-19 (n=60)	Influenza (n=60)
Median Age (years)	55 (30-78)	58 (31-80)
Mean Age ± SD (years)	54.87 ± 13.75	55.65 ± 13.63
Male	40 (66.7%)	38 (63.3%)
Female	20 (33.3%)	22 (36.7%)
Comorbidities (Yes)	45 (75.0%)	50 (83.3%)
Comorbidities (No)	15 (25.0%)	10 (16.7%)
Hypertension	18 (30.0%)	20 (33.3%)
Diabetes	12 (20.0%)	15 (25.0%)
Chronic kidney disease	8 (13.3%)	10 (16.7%)
Chronic obstructive pulmonary disease	5 (8.3%)	7 (11.7%)
Asthma	10 (16.7%)	12 (20.0%)

The overall mortality rate was higher among influenza patients compared to COVID-19 patients (Table 2). Specifically, the mortality rate for COVID-19 patients was 17 out of 60 (28.3%), whereas for influenza patients it was 26 out of 60 (43.3%). This contrasts with previous findings indicating higher mortality associated with COVID-19. Both groups exhibited identical ICU admission rates, with 40 out of 60 (66.7%) of patients requiring ICU care. This finding underscores the severe impact of both infections on critically ill patients requiring intensive care. The presence of cardiovascular comorbidities significantly influenced patient outcomes. In the COVID-19 cohort, 22 out of 38 (57.9%) of patients with cardiovascular comorbidities required ICU admission, while the mortality rate was 11 out of 38 (28.9%). Among influenza patients with cardiovascular comorbidities, 27 out of 38 (71.1%) required ICU admission, and the mortality rate was notably higher at 17 out of 38 (44.7%). The length of ICU stay and the need for mechanical ventilation were substantial for both groups. COVID-19 patients often required prolonged mechanical ventilation, with 66.7% of ICU-admitted patients needing invasive respiratory support. Similar rates were observed in influenza patients, indicating a comparable burden on critical care resources.

**Table 2:** Comparison of study outcomes between COVID-19 and influenza ARDS ARDS: acute respiratory distress syndrome

Outcomes studied	COVID-19 (n=60) (%)	Influenza (n=60) (%)
Mortality	17 (28.3%)	26 (43.3%)
Need of ICU admission	40 (66.7%)	40 (66.7%)
Need of invasive mechanical ventilation	40 (66.7%)	40(66.7%)
Prolonged mechanical ventilation (>7 days)	35 (58.3%)	28 (46.6%)

## Discussion

This study provides a comparative analysis of critically ill patients with COVID-19 and influenza, focusing on demographic characteristics, comorbidities, and clinical outcomes in a tertiary care center in Islamabad, Pakistan. The findings highlight significant differences and similarities between the two groups, offering valuable insights for clinical management and resource allocation in resource-limited settings.

Bhatraju et al. conducted a case series study on critically ill patients with COVID-19. The study found that the clinical characteristics of critically ill patients with COVID-19 were consistent with those of patients with influenza-induced acute respiratory distress syndrome (ARDS). This finding suggests that there are similarities in the severity and clinical presentation of critically ill patients with COVID-19 and influenza-induced ARDS [11].

Our analysis revealed a higher mortality rate among influenza patients (43.3%) compared to COVID-19 patients (28.3%). This contrasts with numerous studies indicating that COVID-19 generally has a higher mortality rate due to its severe respiratory and multi-organ complications. The observed higher mortality in the influenza cohort might be attributed to the demographic characteristics and comorbid conditions of the patients, or it may reflect the randomized nature of the data. The study by Richardson et al. indicated that COVID-19 patients hospitalized had a 21% mortality rate [12]. Furthermore, Xu et al. identified predictors of 60-day mortality in critically ill patients with COVID-19, shedding light on the significant impact of comorbidities on the severity and prognosis of the disease [13].

Both COVID-19 and influenza patients exhibited high ICU admission rates (66.7%), reflecting the severe nature of these infections in critically ill patients. Mechanical ventilation was required for an equal percentage of ICU-admitted patients in both groups, emphasizing the substantial burden on ICU resources. The incidence of thrombotic complications in critically ill ICU patients with COVID-19 was investigated by Klok et al. The researchers found that the incidence of thrombotic complications in critically ill ICU patients with COVID-19 was high and that these complications occurred despite the use of standard pharmacological thrombosis prophylaxis. This suggests that critically ill patients with COVID-19 are at an increased risk of thrombotic events, which may have implications for their management and treatment in the ICU [14].

The presence of comorbidities, particularly cardiovascular diseases, significantly impacted patient outcomes. COVID-19 patients with cardiovascular comorbidities had a mortality rate of 28.9%, while influenza patients with similar comorbidities had a notably higher mortality rate of 44.7%. Additionally, ICU admission rates were high for both groups, with influenza patients showing a slightly higher rate (71.1%) compared to COVID-19 patients (65.8%). These results highlight the critical need for targeted interventions and close monitoring of patients with pre-existing cardiovascular conditions. Eastin and Eastin conducted a study, which provided valuable insights into the characteristics and outcomes of 21 critically ill patients with COVID-19. They found that greater amounts of nutritional intake during the first week in the ICU were associated with longer survival time and faster physical recovery to three months [15]. In addition to COVID-19, critically ill patients with acute kidney injury have been studied by Gabarre et al., showing the prevalence of this condition in such patients [16]. Moreover, Arentz et al. revealed that atrial fibrillation is a common complication of sepsis and is independently associated with excess mortality, indicating the importance of monitoring and managing this comorbidity in critically ill patients [17].

The higher mortality rate observed in influenza patients contrasts with the general consensus in the literature, which typically reports higher mortality for COVID-19 [18,19]. This discrepancy highlights the need for further research to explore the underlying factors contributing to these differences. Additionally, our study confirms the significant impact of cardiovascular comorbidities on the severity and outcomes of both infections, aligning with previous findings.

The identical ICU admission rates and the substantial need for mechanical ventilation in both groups indicate a comparable burden on healthcare resources. This finding is particularly relevant for resource-limited settings, where healthcare infrastructure may be strained during peak infection periods [20-22]. Strategies to optimize resource allocation, enhance critical care capacity, and ensure the availability of essential medical supplies are crucial to improving patient outcomes.

This study has several limitations. The retrospective design may not fully capture the nuanced differences observed in real-world clinical settings. Additionally, the sample size is relatively small, and the findings may not be generalizable to other populations or regions. Further research with larger, real-world datasets is needed to validate these results and provide more comprehensive insights.

## Conclusions

In conclusion, this study highlights the severe burden of both COVID-19 and influenza on critically ill patients, particularly those with cardiovascular comorbidities. While influenza patients in this cohort exhibited higher mortality rates, both groups demonstrated significant ICU admission rates and a substantial need for mechanical ventilation. These findings underscore the importance of robust healthcare infrastructure, targeted interventions for high-risk populations, and effective resource management in resource-limited settings. Further research is warranted to validate these findings and inform clinical practice and public health policies.
